# The Project ENABLE Cornerstone randomized controlled trial: study protocol for a lay navigator-led, early palliative care coaching intervention for African American and rural-dwelling advanced cancer family caregivers

**DOI:** 10.1186/s13063-022-06305-w

**Published:** 2022-06-02

**Authors:** Avery C. Bechthold, Andres Azuero, Maria Pisu, Jennifer Young Pierce, Grant R. Williams, Richard A. Taylor, Rachel Wells, Kayleigh Curry, Rhiannon D. Reed, Erin R. Harrell, Shena Gazaway, Sarah Mollman, Sally Engler, Frank Puga, Marie A. Bakitas, J. Nicholas Dionne-Odom

**Affiliations:** 1 School of Nursing, University of Alabama at Birmingham (UAB), Birmingham, AL USA; 2 Division of Preventive Medicine, UAB School of Medicine, Birmingham, AL USA; 3 USA Health Mitchell Cancer Institute, University of South Alabama, Mobile, AL USA; 4 Division of Hematology and Oncology, Department of Medicine, UAB, Birmingham, AL USA; 5 Division of Transplantation, Department of Surgery, UAB, Birmingham, AL USA; 6 Department of Psychology, University of Alabama, Tuscaloosa, AL USA; 7 College of Nursing, South Dakota State University, Rapid City, SD USA; 8 Division of Geriatrics, Gerontology, and Palliative Care, UAB Department of Medicine, Birmingham, AL USA

**Keywords:** African Americans, Cancer, Family caregivers, Palliative care, Randomized controlled trial, Rural, Telehealth

## Abstract

**Background:**

Family caregivers play a vital, yet stressful role in managing the healthcare needs and optimizing the quality of life of patients with advanced cancer, from the time they are newly diagnosed until end of life. While early telehealth palliative care has been found to effectively support family caregivers, little work has focused on historically under-resourced populations, particularly African American and rural-dwelling individuals. To address this need, we developed and are currently testing Project ENABLE (*E*ducate, *N*urture, *A*dvise, *B*efore *L*ife *E*nds) Cornerstone, a lay navigator-led, early palliative care coaching intervention for family caregivers of African American and rural-dwelling patients with newly diagnosed advanced cancer.

**Methods:**

This is a 2-site, single-blind, hybrid type I implementation-effectiveness trial of the Cornerstone intervention versus usual care. Cornerstone is a multicomponent intervention based on Pearlin’s Stress-Health Process Model where African American and/or rural-dwelling family caregivers of patients with newly diagnosed advanced cancer (target sample size = 294 dyads) are paired with a lay navigator coach and receive a series of six, brief 20–60-min telehealth sessions focused on stress management and coping, caregiving skills, getting help, self-care, and preparing for the future/advance care planning. Subsequent to core sessions, caregivers receive monthly follow-up indefinitely until the patient’s death. Caregiver and patient outcomes are collected at baseline and every 12 weeks until the patient’s death (primary outcome: caregiver distress at 24 weeks; secondary outcomes: caregiver: quality of life and burden; patient: distress, quality of life, and healthcare utilization). Implementation costs and the intervention cost effectiveness are also being evaluated.

**Discussion:**

Should this intervention demonstrate efficacy, it would yield an implementation-ready model of early palliative care support for under-resourced family caregivers. A key design principle that has centrally informed the Cornerstone intervention is that every caregiving situation is unique and each caregiver faces distinct challenges that cannot be addressed using a one-size-fits all approach. Hence, Cornerstone employs culturally savvy lay navigator coaches who are trained to establish a strong, therapeutic alliance with participants and tailor their coaching to a diverse range of individual circumstances.

**Trial registration:**

ClinicalTrials.gov NCT04318886. Registered on 20 March, 2020.

**Supplementary Information:**

The online version contains supplementary material available at 10.1186/s13063-022-06305-w.

## Background

Many of the 2.8 million family caregivers (FCGs) of persons with advanced cancer are historically under-resourced [1], particularly African Americans and rural-dwelling individuals [2]. Both African American and rural-dwelling caregivers report less paid and unpaid assistance with providing day-to-day care and higher hours of care, including more hours providing transportation, advocating for patient needs, and completing medical and nursing tasks [2]. Many have poor access and awareness of formal support services and receive no formal training [1, 3–7]. This is concerning in an advanced cancer context where FCGs provide complex care and support, including managing and monitoring symptoms, coordinating care and communication among multiple specialist providers, managing medications and breathing treatments, giving emotional, spiritual, and companionship support, and providing end of life care [6, 8]. Providing this complex care in combination with coping with a close friend or family member having advanced cancer can be extraordinarily stressful, particularly as individuals approach end of life [9–11]. Patients receiving support from distressed and unprepared FCGs may result in suboptimal home care leading to poorer patient quality of life and increased healthcare utilization [12–14].

Hence, there is a critical need to develop and test interventions for African American and rural-dwelling FCGs [15, 16]. Reports from National Cancer Institute and National Institute of Nursing Research caregiving summits [17, 18], systematic reviews [15, 16, 19], and the National Academy of Medicine [20] have detailed major limitations of existing cancer caregiver interventions, including a lack of attention towards under-resourced populations, unknown implementation costs, questionable scalability, over-reliance on highly trained professionals (e.g., nurses, psychologists, behavioral therapists), lengthy sessions over a short duration, and lack of demonstrated impact on patient outcomes and healthcare utilization. Recognizing these priority areas, we developed and successfully piloted ENABLE (*E*ducate, *N*urture, *A*dvise, *B*efore *L*ife *E*nds) Cornerstone, a lay navigator-led, telehealth-based early palliative care intervention for rural and African American caregivers of patients with newly diagnosed advanced cancer [21, 22]. Building upon our prior trials and community stakeholder formative evaluation work [5, 12, 23–28], this multicomponent intervention is based on Pearlin’s Stress-Health Process Model [29] and consists of a series of six weekly semi-structured coaching sessions and long-term, monthly follow-up until the patient’s death and into bereavement. Lay navigators, overseen by an interdisciplinary outpatient palliative care team, employ health coaching techniques and caregiver distress screening to behaviorally activate and reinforce coaching on managing stress and coping, getting, and asking for help, improving caregiving skills, and decision-making/advance care planning during brief in-person/telephonic sessions plus monthly follow-up from diagnosis through early bereavement [21].

The original ENABLE early palliative care caregiver intervention was tested in a New England population of advanced cancer caregivers [23, 24]. While these results showed that intervention group caregivers had lower depressive symptoms and stress burden, the trial sample included nearly all white individuals and the intervention was led by advanced practice nurse coaches. To adapt this early palliative care intervention to a more historically under-resourced and racially diverse population and leverage a more available workforce, we completed a qualitative formative evaluation study with caregivers and patients of rural and minority populations in the Southern U.S. employing lay oncology navigators [28]. After eliciting and incorporating participant feedback, a new version of the ENABLE Caregiver intervention was developed called ENABLE Cornerstone. ENABLE Cornerstone was subsequently evaluated for feasibility, acceptability, and potential efficacy in a small-scale, pilot randomized trial (intervention vs. usual care) with 63 African American and/or rural-dwelling FCGs (November 2019 to March 2021) [22]. This pilot demonstrated high acceptability and data collection completion rates. In addition, the preliminary efficacy scores for mitigating distress were promising for both Cornerstone caregiver participants and their care recipients. Hence, we are now conducting a fully powered randomized trial of the Cornerstone intervention. This manuscript describes the conceptual foundations of Cornerstone and the clinical trial design.

## Methods and design

### Study aims and hypotheses

The first aim of this hybrid type I randomized implementation-effectiveness trial is to test the effect of ENABLE Cornerstone on FCG outcomes over 24 weeks [30, 31]. Our primary hypothesis is that compared to usual care, intervention group caregivers will report lower distress as measured by the Hospital Anxiety and Depression Scale (HADS) over 24 weeks [32, 33]. Our secondary hypotheses are that compared to usual care, intervention group participants will report better quality of life (QOL) (PROMIS Global Health 10) and lower caregiver burden (Montgomery-Borgatta Burden Scale) over 24 weeks. The second aim is to test the effect of ENABLE Cornerstone on patient outcomes. We hypothesize that compared to usual care, patients of caregivers randomized to receive Cornerstone will report lower distress (HADS), better QOL (PROMIS Global Health 10), and lower healthcare utilization over 24 weeks [34, 35]. For our third aim, we are evaluating implementation costs and the cost effectiveness of Cornerstone implementation on caregiver and patient outcomes, including healthcare utilization. Finally, we have an exploratory aim to analyze mediators and moderators (e.g., resilience, social support effectiveness, preparedness) of the relationship between the intervention and caregiver and patient outcomes.

### Overview of the study design

**Fig. 1:**
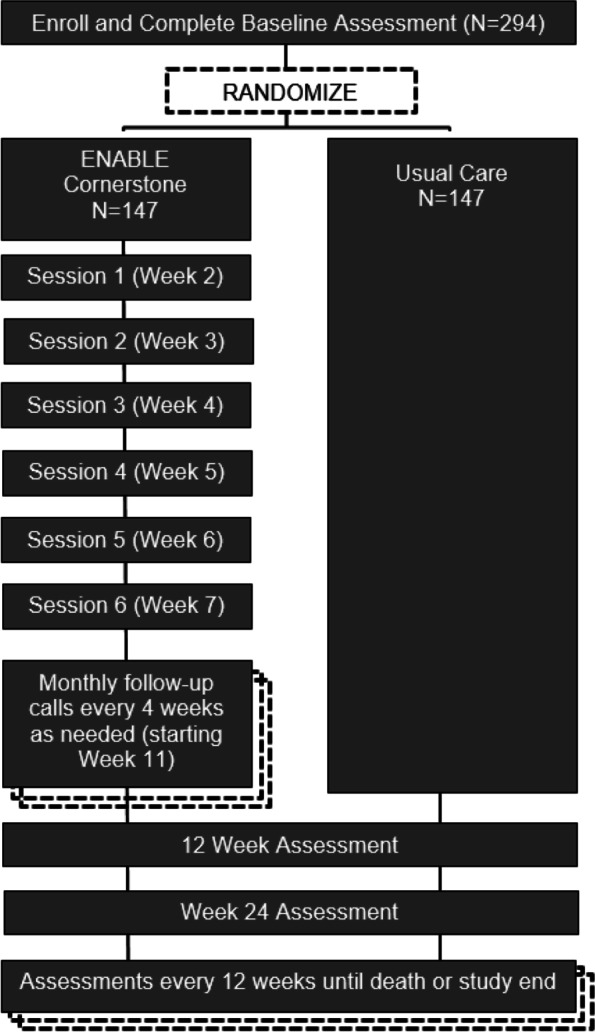
Hybrid type I randomized effectiveness-implementation trial design

This is a 2-site, single-blind, hybrid type I randomized implementation-effectiveness trial comparing the ENABLE Cornerstone intervention to usual care. The target sample is 294 caregivers and 294 of their African American and/or rural-dwelling care recipients with newly diagnosed advanced stage cancer (see Fig. 1). Half of the caregiver participants (*n* = 147) are being randomized to the ENABLE Cornerstone intervention, which is led by a specially trained lay navigator coach and consists of a series of 6 weekly semi-structured coaching sessions and monthly follow-up. The other half of the participants (*n* = 147) are assigned to usual care. Blinded assessments are completed by mailed paper-and-pencil questionnaires at baseline and every 12 weeks until the patient’s death or the study ends. The research protocol was approved by the University of Alabama at Birmingham (UAB) Institutional Review Board (IRB) (#300005045) and the WCG IRB (#20201135) (commercial IRB). The trial is registered as NCT04318886 on clinicaltrials.gov. The study protocol uses the SPIRIT reporting guidelines [36].

### Setting and eligibility criteria

Participants are being recruited from oncology outpatient clinics at two sites: the O’Neal Comprehensive Cancer Center at UAB in Birmingham, Alabama, and the USA Health Mitchell Cancer Institute (MCI) at the University of South Alabama (USA) in Mobile, Alabama.

For FCGs, inclusion criteria are (1) 21 years of age or older; (2) self-endorsing or identified by the patient as “an unpaid spouse/partner, relative, or friend who knows them well and who provides regular support due to their cancer and who does not have to live in the same dwelling”; (3) caring for a patient with an advanced stage cancer; (4) English-speaking; and (5) able to complete baseline measures. Caregiver participants are not required to have an agreeable patient willing to participate in the study. Exclusion criteria include self-reported active severe mental illness (i.e., schizophrenia, bipolar disorder, major depressive disorder), dementia, active suicidal ideation, uncorrected hearing loss, or active substance abuse.

Patients are invited but not required to participate in the trial for data collection purposes only. Patient inclusion criteria are (1) 21 years of age or older; (2) diagnosed within the past 60 days of initial screening with an advanced cancer, defined as metastatic and/or recurrent or progressive stage III/IV cancer, including brain, lung, breast, gynecologic, head and neck, gastrointestinal, genitourinary cancer, and hematologic malignancies; (3) English-speaking; and (4) able to complete baseline measures. Exclusion criteria include (1) receiving hospice and (2) medical record documentation or self-report of active severe mental illness (i.e., schizophrenia, bipolar disorder, or major depressive disorder), dementia, active suicidal ideation, uncorrected hearing loss, or active substance abuse.

### Recruitment

**Fig. 2:**
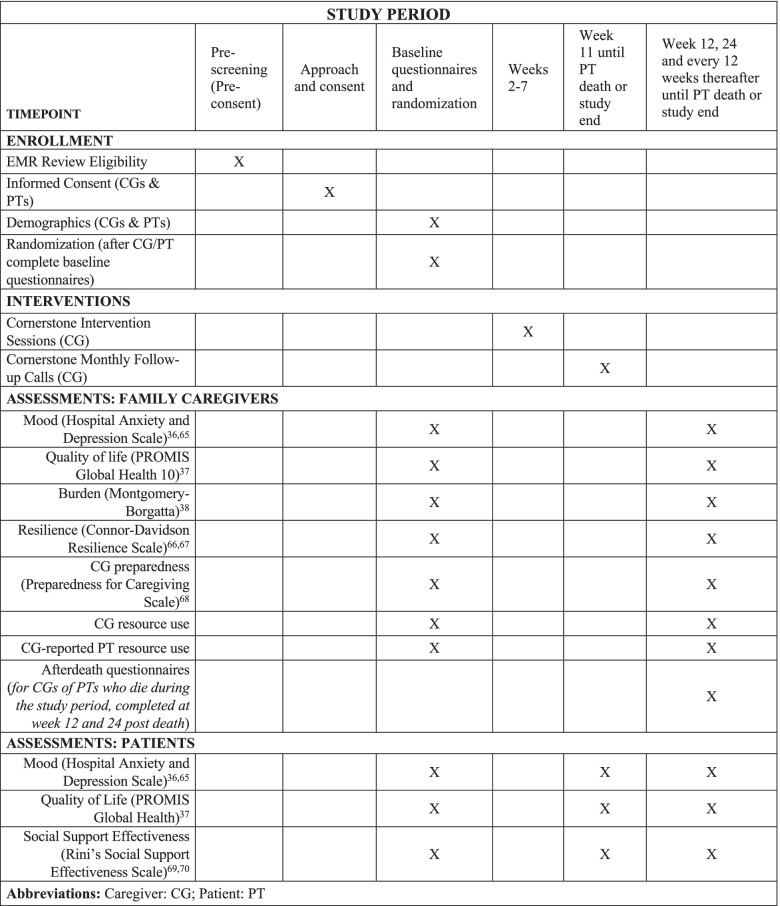
SPIRIT figure of study enrollment, interventions, and assessments

Our recruitment approach is clinic-based but executed remotely. Research coordinators access the electronic medical record and screen for initial patient eligibility by reviewing the outpatient clinic schedules of partnering oncologists. All patients with a planned office visit in the upcoming 1 to 2 weeks are reviewed for eligibility. For patients appearing to meet eligibility criteria, an opt-out email is sent to the patient’s oncologist, which informs them that the individual will be approached for participation in the study and to reply to the email only if they do not want the patient approached. In our pilot trial [22], recruiters initially approached patients and their FCGs in-person prior to their appointment in the waiting room to introduce the study and invite eligible and agreeable individuals to participate. However, COVID-19 forced us to shift from in-person recruitment to a remote approach. For the rest of our pilot trial and for this current trial, study coordinators mail a study flyer and an opt-out letter the day of the patient’s clinic appointment (arriving at their home a few days later) to introduce the study and let them know that a member of the research team will be contacting them by phone to discuss the study. Individuals are given a number to call if they do not wish to be contacted further for the study. A recruiter then calls the participants by phone to introduce the study, assess eligibility, and invite them to participate. Participants are consented verbally via an IRB approved waiver of *signed* informed consent (caregiver and patient consent form templates available upon request). Consented participants are immediately mailed a copy of the informed consent document for their keeping and a paper-and-pencil baseline survey to complete and return by mail. Once the study team receives these completed baseline surveys in the mail, the caregiver and patient (if participating) are randomized. See Fig. 2 for a SPIRIT figure.

### Randomization and blinding

Randomization occurs at the level of the caregiver participant, with participating patients assigned to the same group as their caregiver. The randomization scheme is executed via REDCap (Research Electronic Data Capture) [37], a secure web-based software platform designed to support randomization and data capture for research studies. Participants are randomly assigned (1:1) using a computer-generated program overseen by the trial statistician. The randomization scheme is stratified by site (UAB and MCI) and blocked within strata (using random block lengths 4 and 10). After randomizing a FCG in REDCap and if selected for the Cornerstone program, the assigned lay navigator informs them by phone of their group assignment and introduces the intervention activities. If randomized to the usual care group, the program manager notifies FCGs by mail of their group assignment. All other members of the research team, including the principal investigator (PI) and all co-investigators, are blind to group assignment and participants are instructed to not discuss their assignment with study staff. The allocation sequence will remain concealed until the last participant completes 24-week data collection and the data have been checked for completeness and accuracy.

### The ENABLE Cornerstone Intervention

Caregivers randomized to the intervention condition are paired with a specially trained lay navigator coach. The lay navigator coach’s roles are to (1) provide basic psychoeducation on relevant caregiving topics; (2) offer health coaching and problem-solving support for problems and self-care goals identified by caregivers; (3) perform caregiver distress screening and support; (4) bridge the communication gap between the healthcare team and the family and patient as needed; (5) provide families with the appropriate cancer-specific institutional, local, state, and national resources; (6) offer basic psychological and emotional support; and (7) serve as a continuity figure for the caregiver during the patient’s trajectory of serious illness, from diagnosis of advanced disease to bereavement.

After contacting the participant by phone, the coach and caregiver proceed with scheduling six weekly coaching sessions. Coaching sessions can be conducted in-person at a mutually agreed upon location (including the participant’s home) or over the phone. However, all sessions have been conducted over the phone since the emergence of COVID-19 in March 2020 to the date of this publication. Core coaching sessions cover specific caregiver topics identified from our formative evaluation study (Table 1) [28]. Sessions are designed to last approximately 20 min but may last up to 1 h if desired by the participant. The six sessions are followed by monthly follow-up calls that focus on monitoring caregivers’ distress (via distress screening), reinforcing content covered in core sessions, providing additional informational materials, and initiating referrals for additional support. For caregivers of patients who die, coaches conduct a bereavement call approximately 2 to 6 weeks after the person’s death to acknowledge and express sympathy for the caregiver’s loss and to provide and review additional bereavement materials and resources.

**Table 1:** ENABLE cornerstone core sessions, monthly follow-up, and bereavement call

Core session	Objective	Content
Session 1: Caregiving story	Establish therapeutic alliance by exploring and validating the individual’s caregiving situation	- Supporting someone with cancer - National caregiving statistics and commonly experienced challenges - Understanding your caregiving experience, including biggest current and future concerns and what gives you strength -Orientation to and administration of caregiving distress thermometer
Session 2: Coping with stress	Introduce caregivers to the stress process model and discuss ways to cope with stress	- Distress screening - How stress works - Ways to cope with stress - Action plan for the coming week
Session 3: Getting help	Motivate effective social support through asking for and getting help	- Distress screening - Why some families do not ask for help - Getting help from family, friends, and community resources - 3 options for accomplishing caregiving tasks: doing it yourself, asking for volunteers (e.g., other family members and friends), and paying for help - How to decide what to take on yourself and when to ask others for help - Action plan for the coming week
Session 4: Improving your support skills	Enhance caregiving skills and organization	- Distress screening - Tips for organizing health information, managing medications, and tracking symptoms - Providing your loved one the “right” amount and type of support - Action plan for the coming week
Session 5: Taking care of yourself	Improve and reinforce self-care behaviors	- Distress screening - Maintaining health while under stress - Completing a self-care inventory and developing a personal health plan - Action plan for the coming week
Session 6: Decision-making and planning for the future	Help develop plans for the future to help mitigate future stressors and potential crises	- Distress screening - Partnering with patients to make decisions in serious illness - Making decisions about cancer treatment, advance care planning, and advance directives - Basic principles of communication when making decisions - Action plan for the coming month
Monthly follow-up (every 4 weeks)	Ensure continuity of care, conduct caregiver distress screening, and reinforce content covered in core sessions	- Distress screening - Additional informational materials and/or initiate referrals for additional support
Bereavement call (2–6 weeks post death)	Acknowledge and express sympathy for the caregivers loss and review resources for bereavement support	- Additional informational materials and/or initiate referrals for additional bereavement support - Closure of coaching relationship

Each core session and monthly follow-up encounter begins with a caregiver distress thermometer screening, using a thermometer tool adapted in our prior work from the National Comprehensive Cancer Network Patient Distress Thermometer [21, 38, 39]. Details on the thermometer tool design and administration are reported elsewhere [39]. After identifying a problem, the coach can offer four types of support: (1) information and educational materials that have been pre-selected and reviewed by overseeing clinicians (RAT, RW, JP, GW, JND-O); (2) problem-solving support to help the participant work towards a longer-term goal to manage the problem [27]; (3) health coaching to help the participant work towards enhancing a focused area of self-care; and (4) referral to another professional, formal service, or community group. Distress screenings also help the coach to personalize session discussions to the caregiver’s specific needs and challenges.

Based on feedback from our pilot, an action planning exercise was added to the end of each core session, based on the steps and principles of motivational interviewing [40]. Caregivers are asked to name one or two things they would like to get better at based on what was discussed during the session. If a goal behavior or action is identified, the coach initiates action planning support to guide the caregiver in assessing why this goal matters to them, what their confidence is in achieving the goal, and what concrete steps are needed in the coming week to attain the goal. Coaches begin all subsequent sessions and monthly follow-up calls with an inquiry about the participant’s progress on prior weeks’ action plan goals, including celebrating successes, reflecting on challenges, and helping them assess next steps (for the coming week) to continue to make progress towards their goal.

Prior to the first core session, intervention group caregivers are mailed a study team-developed Project ENABLE Cornerstone Toolkit. This 3-ring, self-enclosed binder contains educational information pertaining to the six core sessions and serves as an all-in-one organizational binder for intervention materials and resources (i.e., business card holders; FCG tracking sheets for patient medications, tests, and procedures; and a calendar).

#### Conceptual basis of ENABLE Cornerstone

**Fig. 3:**
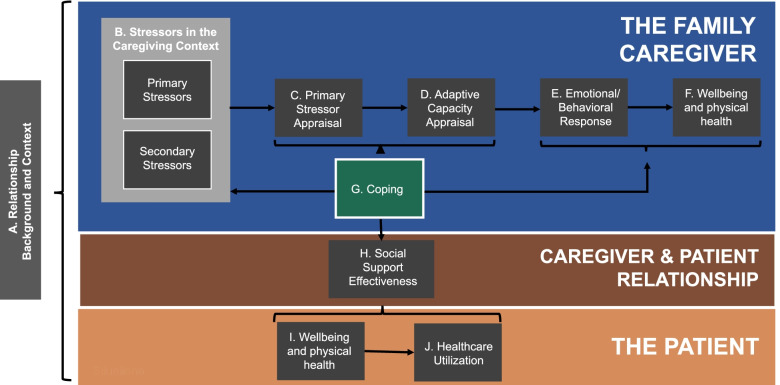
Adapted Pearlin’s Stress-Health Process Model of Family Caregiving

**Fig. 4:**
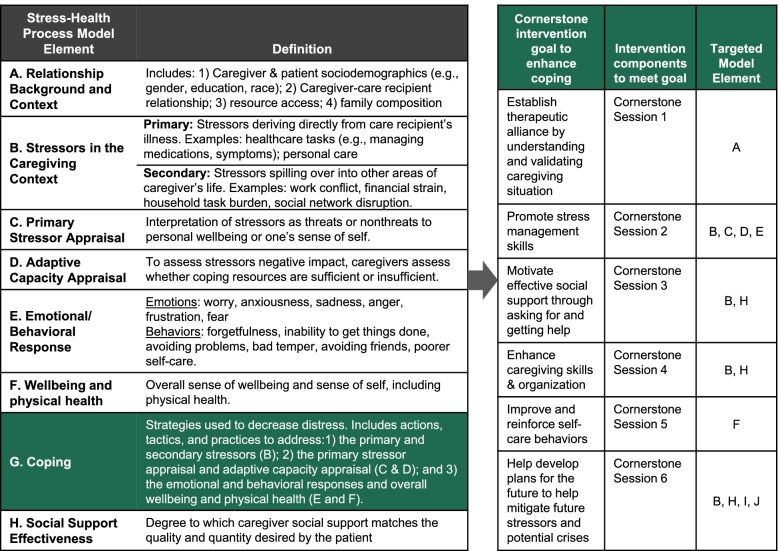
Stress-Health Process Elements and Cornerstone Components Targeting those Elements

The ENABLE Cornerstone intervention is conceptually based on our team’s adapted version of Pearlin’s Stress-Health Process Model (Fig. 3) [29], which consists of three primary domains: the FCG, the caregiver and patient relationship, and the patient. Within these domains are specific key constructs, each preceded by a letter. In Fig. 4, definitions of these key constructs are shown on the left panel, while on the right panel, we link each Cornerstone session with constructs in the model they are designed to enhance. Cornerstone ultimately seeks to reduce stressors (B), enhance the caregiver’s capacity to appraise stressors (C, D), and change caregivers’ negative emotional and behavioral responses (E) by enhancing their coping skills (G). We believe this should subsequently decrease overall distress for the caregiver (F) and enhance their ability to provide high-quality social support to the patient (H). This would thereby optimize the patient’s quality of life (I) and their healthcare utilization (J).

#### Discontinuation of study intervention

Participants may withdraw from the study or voluntarily stop intervention sessions at any time. Reasons for discontinuing the intervention are documented (e.g., too burdensome, too busy, patient too ill), though the participant is invited to continue with data collection. The PI can also discontinue a participant from the study for the following reasons: (1) significant study intervention non-adherence, (2) lost to follow-up, or (3) occurrence of an event, medical condition, or situation such that continued collection of study data would not be in the best interest of the participant.

### Usual care

Guided by NIH consensus panel recommendations [41], we determined that a usual care condition was the optimal comparator to best serve the aims of this trial. Usual care at both the UAB and MCI sites consists of resources focused primarily on the patient; no specific caregiver services exist to support their unique needs. A usual care comparison will test whether caregivers experiencing distress need more active support than what is currently offered by traditional services. Immediately after randomization, participants in both groups are mailed pamphlets outlining UAB and MCI resources for families. To further examine and describe usual care [42], we are collecting information from participants in both conditions on whether patients and/or FCGs on behalf of patients accessed UAB or MCI resources for families.

### Interventionist training and oversight

To facilitate future replication and dissemination of ENABLE Cornerstone, should it demonstrate efficacy, our team has developed a structured orientation and training curriculum to prepare lay navigator coaches, based on our team’s lay navigator expertise [43–45] and training approaches from our prior trials [21, 22, 24, 46]. The training program (see Table 2) consists of 10 modules (~70 h total) housed on a web-based education platform called Canvas. Each module includes independent readings, videos demonstrating coaching techniques (e.g., active listening, single- and double-sided reflections, action planning), for all study protocols, procedures, and scripts, example session recordings, and one-on-one role-playing practice for each of the six sessions with a standardized caregiver.

**Table 2:** Description of project cornerstone RCT coach training

Module	Title	Description
1	Before you get started	Access and orientation to study files and tracking.
2	Fundamentals of supportive care coaching	Cover fundamentals of oncology navigation, health coaching, family caregiving, and palliative care in oncology.
3	What is this study about?	Review background on Project Cornerstone and protocols of the current trial.
4	Cornerstone Session 1: Caregiving story	Establish rapport with caregivers, orient them to the Toolkit, ask them about their day-to-day lives, and administer distress screening.
5	Cornerstone Session 2: Coping with stress	Educate caregivers about the stress process model and how to cope with stress.
6	Cornerstone Session 3: Getting help	Review why caregivers do not ask for help and how to accomplish caregiving tasks by leveraging outside help.
7	Cornerstone Session 4: Caregiving skills	Review tips for organizing health information and medications and how to track symptoms; provide guidance on how to communicate with care recipients about the type and amount of support they desire.
8	Cornerstone Session 5: Self-care	Review and discuss self-care and facilitate a comprehensive self-care assessment with family member participants.
9	Cornerstone Session 6: Decision-making and advance care planning	Discuss decision-making in serious illness and the role of family members.
10	Monthly follow-up, bereavement, and suicide protocol	Learn how to check in with family caregivers monthly, provide condolences and support after a patient’s death, and act if a family member or patient expresses a desire to harm themself or others.

Throughout the study, lay navigator coaches engage in weekly supervisory meetings with the PI (a board-certified palliative care advanced practice nurse [JND-O]) and a nurse practitioner co-investigator (RAT) to review calls and sessions with all active intervention group participants, including adverse events. In addition, coaches have 24/7 access to the PI for any acute participant issues that arise.

### Treatment fidelity monitoring

Four strategies (consistent with NIH and the Template for Intervention Description and Replication [TIDieR] guidelines) [47, 48] are being employed to ensure intervention fidelity. First, as described above, there is structured and standardized training for all interventionists. Second, interventionists use guiding scripts for each of the core sessions and monthly follow-up calls. Third, charting templates are used by interventionists for each intervention contact to further ensure that all materials are covered. Finally, all core sessions are audio recorded by coaches and 25% are randomly selected on a quarterly basis for treatment fidelity monitoring by a study team member using a fidelity checklist. If ratings for a coach fall below 80% adherence, retraining in underperforming areas will be overseen by the PI.

### Data collection and measures

All measures for this study were selected to assess distinct constructs in our Adapted Pearlin’s Stress-Health Process Model of Family Caregiving (Fig. 3) and are listed and described in Table S1 (see Additional file 1). Measures are collected at baseline and every 12 weeks thereafter until the patient’s death or study end (data collection forms available upon request). For each time point, paper-and-pencil questionnaires are mailed to participants for them to complete and return in a prepaid, preaddressed envelope. Participants are subsequently paid via a Mastercard debit card at an escalating incentive amount per time point to enhance retention: $40 for baseline, $50 for 12 weeks, and $60 for 24 weeks. For every 12-week time point after that, participants receive $30.

After participants complete the questionnaire, surveys are returned in postage paid, preaddressed envelopes where data collection coordinators double-key enter de-identified survey responses into REDCap. REDCap is software for building and managing questionnaires and facilitating electronic data collection and storage [37]. It supports a HIPAA best practice, secure web-based application. Hard stops prevent missing data due to inadvertent skipping of items. This database is accessed on a secure network server that is password protected. Descriptive statistics will be used to conduct quality control on the preliminary datasets and identify missing/extreme data values. Study coordinators who have received intensive training (typically 10 h including role play and inter-rater reliability checks) will assure high-quality data collection from participants.

### Safety monitoring committee

This is a two-site study that has been deemed low risk, and hence, there are no planned interim analyses and a Data Safety and Monitoring Board is not appointed. However, the informed consent process, the recruitment process, and the timeliness and quality of the data will be monitored by the principal investigator, the WIRB Institutional Review Board, and a Safety Monitoring Committee (SMC). The SMC provides safety oversight and annual auditing and is composed of individuals with clinical trial, human subjects, and statistical expertise. The two co-chairs of the SMC are independent from study conduct and free of conflict of interest. The SMC meets annually to assess effectiveness data from each arm of the study (blinding is maintained). A SMC report is provided to the IRB on an annual basis.

### Statistical analysis plan

An intention-to-treat (ITT) approach will be used for analyses of Aims 1 and 2. Primary data analysis will begin with baseline descriptive statistics for baseline caregiver and patient characteristics and outcomes. The balance between study groups will be examined with respect to baseline characteristics using effect sizes. Conceptually relevant baseline factors showing non-trivial imbalances between groups will be used as adjusting covariates in longitudinal comparisons using linear mixed-effect modeling. Distributional assumptions will be examined, and when appropriate, we will employ inferential and modeling procedures robust to distributional assumptions.

#### Power analysis and sample size determination

Key considerations in our power analysis included (1) longitudinal modeling [49] with baseline and two follow-up time points (weeks 12 and 24 post-baseline); (2) 80% power to detect a mean difference in change; (3) a significance level adjusted with a false discovery rate approach (5% FDR) with relevant effects on 50% of outcomes, i.e., *α* = .025; (4) intra-subject correlation of *ρ* = .5 among repeated measurements; (5) minimal clinically important difference (MCID) for the primary outcomes, HADS-Anxiety and HADS-Depression scales of 1.5 points estimated from an adult population with psychological distress but no chronic disease, such as caregivers; and (6) standardized MCID of *d* = .37, using the HADS standard deviations (SD~3.9) from our prior Cornerstone study. Under these assumptions, with a sample size of *n* = 103 per study group, the detectable difference is *d* ≈ .37; however, a sample size of *n* = 120 per group allows additional power and precision for secondary analyses including comparisons of patient outcomes and exploratory analyses. Target recruitment is *N* = 294 FCGs (*n* = 147 per study group) to account for a possible dropout of up to 37.7% observed among caregivers enrolled in one of our prior trials (computations conducted with PASS v14 software).

### Dissemination plans

Results will be submitted for peer-reviewed publication. After completion of all analyses, data will be made available upon request to the PI.

## Discussion

We are implementing a type I hybrid implementation-effectiveness trial to test the benefit and evaluate the costs of ENABLE Cornerstone, an early palliative care intervention for under-resourced African American and rural-dwelling FCGs of patients with newly diagnosed advanced cancer. This problem is widely recognized as a public health crisis [20, 50, 51] and a nursing and palliative care priority [52–54]. Our prior developmental and testing work [28] has highlighted a key design principle that has centrally informed the Cornerstone intervention: every caregiving situation is unique and each caregiver faces distinct sets of challenges that cannot be addressed using a one-size-fits all approach. Hence, Cornerstone has been designed as a multicomponent package of intervention components that continuously assess the caregiver’s unique circumstances and is equipped to address multiple sources of acute and future caregiving distress.

A second implication of this design principle is the features of Cornerstone that are intended to promote a strong, trusting relationship between the interventionist and the caregiver. Research on therapeutic alliance has shown that the relationship between a therapist and a client can itself be one of the primary determinants of positive behavior change and outcomes [55–57]. For Cornerstone, the lay navigator as a health coach establishes an equal partnership, where they guide the reflection and skills enhancement process, and caregivers are the guides and experts on their own lives, values, and the patient’s health status and goals [58]. Coaches are trained to have a deep respect for the caregiver’s autonomy and an understanding that the problems, goals, and action steps are chosen by caregivers according to their needs and values. We believe this coaching partnership feature is particularly important in a context when the caregiver is from a historically excluded, marginalized, and under-resourced community, and can be distrusting towards healthcare institutions.

To further reinforce the relationship between coaches and caregivers, Cornerstone employs lay navigators. To date, lay healthcare navigators have typically provided one-on-one guidance to patients and families in navigating insurance and financial issues, treatments and healthcare options, basic emotional support, transportation, patient appointments, and communicating with the healthcare team [59, 60]. Although they often lack a formal healthcare background, lay navigators are respected, trusted, and embedded within the community (cultural in-group members), which makes them ideal for working with under-resourced populations [59, 60]. For Cornerstone, lay navigators receive additional training as coaches to facilitate psychoeducation in caregiving and palliative care principles, goal setting and behavioral activation, and psychological and emotional support. Furthermore, if Cornerstone is efficacious in this trial, we anticipate it to be highly scalable, since lay navigation programs have proliferated in cancer centers since their inclusion in the Affordable Care Act [61].

A third implication of the not-one-size-fits-all design is that Cornerstone follows caregivers across the entire serious illness trajectory, from initial diagnosis of advanced cancer through bereavement. Reviews of caregiver interventions have noted relatively short duration and confinement to a single setting or context as key limitations of tested programs [15, 20]. To address this limitation, Cornerstone coaches serve as a continuity figure for FCGs as new challenges arise and as their care recipients inevitably become more disabled and ill over time.

To conclude, we are testing the effect of Project ENABLE Cornerstone on caregiver and patient outcomes and evaluating implementation costs and the cost effectiveness among under-resourced FCGs of both African American and rural-dwelling persons with newly diagnosed advanced cancer. Such an intervention is critically needed for these two populations in the U.S. South which has poor access to palliative care [3, 4]. By leveraging a relatively untapped lay navigator workforce, we have the potential to greatly enhance the reach of specialty palliative care [62].

### Trial status

Western IRB Protocol Number: 20201135; UAB IRB Protocol Number: 300005045. Version Number: V.1.2 (last updated 14 May 2021). Recruitment began in January 2021, and we anticipate completing accrual in June 2025.

## Supplementary Information

**Additional file 1: Table S1.** Aims, Outcomes and Associated Measures [63, 64, 65, 66, 67].

## Data Availability

De-identified quantitative data and codebook will be available from the authors upon request.

## Abbreviations

Co-I: Co-investigator
ENABLE: Educate, Nurture, Advise, Before Life Ends
FDR: False discovery rate
FCG: Family caregiver
HADS: Hospital Anxiety and Depression Scale
ITT: Intention-to-treat
MCI: Mitchell Cancer Institute
MCID: Minimal clinically important difference
PI: Principal Investigator
QOL: Quality of life
RCT: Randomized controlled trial
TIDieR: Template for Intervention Description and Replication
UAB: University of Alabama at Birmingham
US: United States
