# Supplementary material for: The Project ENABLE Cornerstone randomized controlled trial: study protocol for a lay navigator-led, early palliative care coaching intervention for African American and rural-dwelling advanced cancer family caregivers
Source: Trials. 2022 Jun 2;23:452. doi: 10.1186/s13063-022-06305-w (PMC9161197; doi:10.1186/s13063-022-06305-w)
Supplement: Supplementary file 1 — Additional file 1: Table S1. Aims, Outcomes and Associated Measures [63, 64, 65, 66, 67]. [file 13063_2022_6305_MOESM1_ESM.docx]

**Table 1**. Aims, Outcomes and Associated Measures

| **Aims** | **Outcomes** | | | |
| --- | --- | --- | --- | --- |
| Aim 1: Test the effect of ENABLE Cornerstone on caregiver outcomes. | **Outcome: Measure** | **# Items** | **Description** | **Interpretation** |
|  | Distress: HADS (Primary outcome)^32,33^ | 14 | Detects the presence and severity of mild degrees of mood disorder, anxiety, and depression. Consists of two subscales: anxiety (e.g., feeling tense, restless, worry; 7 items) and depressive symptoms (e.g., cheerfulness, feeling slowed down; 7 items). | Higher scores = worse anxiety/ depression |
|  | Health-Related Quality of Life: PROMIS Global Health 10^34^ | 10 | Measures global HRQOL in 2 domains: physical and mental health. Scoring allows for estimates of cost effectiveness. | Higher scores = higher HRQOL |
|  | Burden: Montgomery-Borgatta Caregiver Burden Scale^35^ | 14 | Measures caregiver burden with 3 domains: objective burden, stress burden, demand burden. | Higher scores = higher burden |
| Aim 2: Test the effect of ENABLE Cornerstone on patient outcomes. | Mood: HADS^32,33^ | 14 | *Same as above under Aim 1* | Higher scores = worse anxiety/ depression^35,64^ |
|  | Health-Related Quality of Life: PROMIS Global Health 10^34^ | 10 | *Same as above under Aim 1* | Higher scores = higher HRQOL |
|  | Healthcare Utilization: Resource Use Questionnaire (caregiver-reported) | - | Inpatient days, ICU days, ED visits, hospice use, palliative care provider visits, advance care planning conversation, AD completion, DNR orders. | - |
| Aim 3: Evaluate implementation costs and the cost effectiveness of ENABLE Cornerstone implementation on caregiver and patient outcomes, including healthcare utilization. | Health-Related Quality of Life: PROMIS Global Health 10^34^ | 10 | *Same as above under Aims 1 and 2* | Higher scores = higher HRQOL |
|  | Family Caregiver Resource Utilization | - | Receipt of therapy/counseling; education/training; practical support; spiritual support; national/state/local organization assistance; hours of assistance; FCG support through UAB or MCI cancer centers; outpatient provider visits (self), ED visits (self), hospital/ICU days (self); paid/unpaid time off work; out-of-pocket costs (self & patient). | - |
|  | Healthcare Utilization: Resource Use Questionnaire (caregiver-reported) | - | Same as above under Aim 2 | - |
| Exploratory aim: Explore mediators and moderators of the relationship between the intervention and caregiver and patient outcomes. | **Family caregivers:** | | | |
|  | Resilience: Connor-Davidson Resilience Scale^63,64^ | 10 | Assesses a person’s ability to thrive in adversity; personal competence, tolerance of stress/negative feelings, acceptance of change, ability to bounce back. | - |
|  | Preparedness: Preparedness for Caregiving Scale^65^ | 8 | Measures preparedness for caregiving in 3 domains: providing physical and emotional care, arranging services, & dealing with stress. | - |
|  | **Patients**: | | | |
|  | Social support: Rini’s Social Support Effectiveness Scale^66,67^ | 25 | Measures the amount, quality, and skillfulness of support from friends and relatives, including task, emotional, and informational support subscales. | - |

*Note*. Abbreviations: AD = advance directive; DNR = Do Not Resuscitate; ED = emergency department; ENABLE = Educate, Nurture, Advise, Before Life Ends; HADS = Hospital Anxiety and Depression Scale; HRQOL = health-related quality of life; ICU = intensive care unit; PROMIS = Patient-Reported Outcomes Measurement Information System
